# Effects of 20 Hz Repetitive Transcranial Magnetic Stimulation on Disorders of Consciousness: A Resting-State Electroencephalography Study

**DOI:** 10.1155/2018/5036184

**Published:** 2018-03-25

**Authors:** Fangping He, Min Wu, Fanxia Meng, Yangfan Hu, Jian Gao, Zhongqin Chen, Wangxiao Bao, Kehong Liu, Benyan Luo, Gang Pan

**Affiliations:** ^1^Department of Neurology and Brain Medical Centre, First Affiliated Hospital, School of Medicine, Zhejiang University, Hangzhou 310003, China; ^2^Department of Computer Science, Zhejiang University, Hangzhou 310007, China; ^3^Department of Rehabilitation, Hangzhou Hospital of Zhejiang Armed Police Corps, Hangzhou 310051, China; ^4^Department of Neurology and Brain Medical Centre and Collaborative Innovation Center for Brain Science, First Affiliated Hospital, School of Medicine, Zhejiang University, Hangzhou 310003, China

## Abstract

Repetitive transcranial magnetic stimulation (rTMS) has been proposed as an experimental approach for the treatment of disorders of consciousness (DOC). To date, there has been little research into the use of rTMS in DOC and the therapeutic effects have been variously documented. This study aimed to examine the effects of 20 Hz rTMS on the electroencephalography (EEG) reactivity and clinical response in patients with DOC and to explore the neuromodulatory effects of high-frequency rTMS. In this randomized, sham-controlled, crossover study, real or sham 20 Hz rTMS was applied to the left primary motor cortex (M1) of patients with DOC for 5 consecutive days. Evaluations were blindly performed at the baseline (T0), immediately after the end of the 5 days of treatment (T1) and 1 week after the treatment (T2) using the JFK coma recovery scale-revised (CRS-R) and resting-state EEG. Only one patient, with a history of 2 months of traumatic brain injury, showed long-lasting (T1, T2) behavioral and neurophysiological modifications after the real rTMS stimulation. The 5 remaining patients presented brain reactivity localized at several electrodes, and the EEG modification was not significant. rTMS stimulation may improve awareness and arousal of DOC. Additionally, EEG represents a potential biomarker for the therapeutic efficacy of rTMS. This trial is registered with (NCT03385278).

## 1. Introduction

At present, there are no evidence-based guidelines regarding the awakening treatment of patients with disorders of consciousness (DOC). Neurostimulation techniques hold considerable promise for potential therapeutic intervention. Deep brain stimulation (DBS) has been attempted in a single patient with minimally conscious state (MCS) [1]. Considering the invasiveness and the ethical and procedural limitations of the use of DBS in such patients [2], repetitive transcranial magnetic stimulation (rTMS) has been introduced as a painless and noninvasive alternative approach. Currently, rTMS has emerged as an effective treatment for mental and dyskinetic disorders [3–5].

Transcranial magnetic stimulation (TMS) involves the use of alternating magnetic fields to stimulate neurons in the brain. To date, few studies have been conducted investigating the efficiency of rTMS in patients with impaired consciousness. Manganotti et al. reported that rTMS over the motor cortex induced prolonged (38 min after rTMS) behavioral and neurophysiological modifications in one MCS patient [6]. Piccione et al. reported that a single session of 20 Hz rTMS delivered over the primary motor cortex (M1) induced behavioral improvements and an increase in the EEG power in a MCS patient [7]. However, in a recent study, no therapeutic effect of 20 Hz rTMS at M1 was identified in chronic vegetative state (VS), in a sham-controlled study [8]. Reviewing previous data, the feasibility and safety of high-frequency rTMS in DOC have been confirmed with no reported side effects [9], but the effectiveness has not yet been thoroughly studied. Moreover, only one study has observed cortical activation lasting up to 6 hours at most in response to rTMS in MCS [6]. Currently, no advanced studies have reported longer-lasting modulatory effects of rTMS.

EEG is a widely accepted neurophysiological method for monitoring brain function on the level of cortical information processing and changes that occur during unconsciousness and varying states of conscious awareness [10]. A relationship between responsive alpha activity and conscious awareness has been demonstrated. Furthermore, EEG measurements allow researchers to distinguish the VS from the MCS and help to establish the prognosis of patients [11–14].

Thus, in the current randomized, sham-controlled, crossover study, we aimed to evaluate the short- and long-term efficiency of rTMS in the treatment of DOC. We examined the clinical responses and EEG reactivity in 6 patients with DOC before and after a protocol of 20 Hz rTMS.

## 2. Methods

### 2.1. Patients

Six patients in the Department of Rehabilitation at Hangzhou Hospital of Zhejiang CAPR with severe closed craniocerebral injury who were recovering from a coma (4 men, 2 women; mean age, 39.5 years) were included in our study. All subjects met the study inclusion criteria: (1) no centrally acting drugs; (2) no neuromuscular function blockers and no sedation within the prior 24 hours to the study; (3) periods of spontaneous eye opening (indicating preserved sleep-wake cycles); and (4) a diagnosis of VS, MCS, or emerged from MCS (EMCS) based on the coma recovery scale-revised (CRS-R) [15, 16]. Individuals with contraindication for rTMS and other severe neurological or systemic diseases were excluded [6, 17]. The demographic and clinical characteristics of the enrolled patients are shown in Table 1.

The trial was conducted in accordance with the Declaration of Helsinki. Written informed consent was obtained by the legal representative of each patient. This study was approved by the Ethical Committee of the First Affiliated Hospital, School of Medicine, Zhejiang University and Hangzhou Hospital of Zhejiang CAPR.

### 2.2. Design and Stimulation Procedures

**Figure 1:**
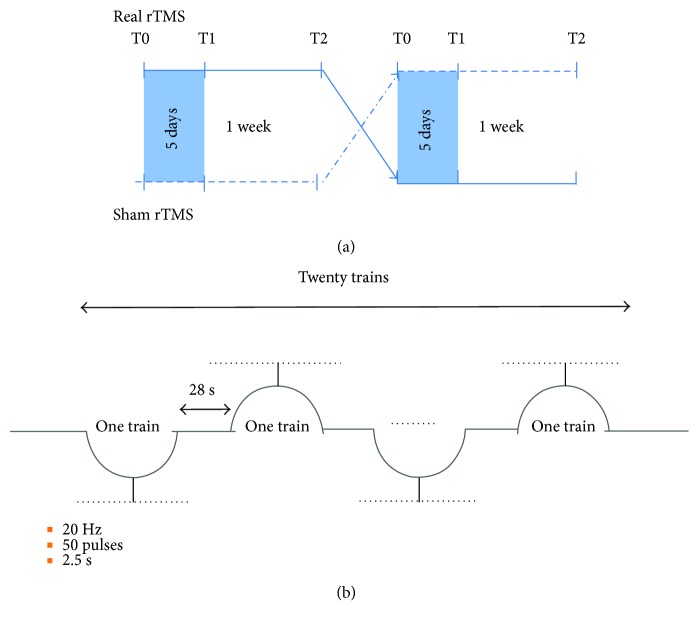
rTMS protocol for the patients. (a) The study design. All patients received active 20 Hz rTMS and sham stimulation for 5 consecutive days over the left primary motor cortex (M1), in separate sessions spaced one week. Clinical evaluation and EEG data were assessed at the baseline (T0), immediately after the end of the 5 days of treatment (T1) and 1 week after the treatment (T2). (b) rTMS procedure. A session of 1000 pulses were delivered in 20 trains. Each stimulation train lasted 2.5 s with a 28 s intertrain pause.

This study was designed as a sham-controlled, randomized, crossover trial and the experiment always initiated between 8 : 00 and 10 : 00 a.m. In the real stimulation session, rTMS was administered over 5 consecutive working days (from Monday to Friday), and in the sham stimulation session, sham rTMS was also administered over 5 consecutive working days (from Monday to Friday). Among the 6 eligible patients, three patients first received real rTMS, and the remaining three patients first received sham stimulation. Participants have a “wash-out” period of 1 week between both sessions (Figure 1). Real rTMS was administered to the scalp over the left M1 with a Magstim-Rapid2 stimulator (Magstim Company Ltd., London, UK) [18]. TMS was delivered through a figure-of-eight focal coil oriented so that the induced electric current flowed in a posterior-anterior direction over the left M1. According to the recommendations of the International Federation of Clinical Neurophysiology Committee [19], stimulation intensity was determined based on the resting motor threshold (RMT) for every subject, which was defined as the minimum TMS intensity that produced a stretch of the right thumb in at least 5 out of 10 consecutive trials during muscle relaxation. rTMS was performed at 20 Hz, at an intensity of 100% of the RMT. If the RMT was over 67%, an intensity of the 60% maximum stimulator output was used in accordance with international safety recommendations. The rTMS procedure consisted of a session of 1000 pulses delivered in 20 trains of 20 Hz with a stimulus intensity equal to RMT. Each stimulation train lasted 2.5 s with a 28 s intertrain pause. Sham stimulation was delivered using the same protocol except that the angled coil was positioned away from the head. Thus, the magnetic field could not penetrate the brain, although the acoustic artefact of real stimulation was reproduced in the sham rTMS [20].

### 2.3. Clinical Evaluation

The experimenters who assessed behavioral improvement were blind to the experimental design. Clinical evaluations were performed at baseline (T0), immediately after the end of 5-day treatment (T1) and 1 week later (T2) with the CRS-R (Table 2). The CRS-R is a tool used to characterize the level of consciousness and to monitor neurobehavioral recovery in DOC [21]. The scale consists of 23 hierarchically arranged items that comprise six subscales addressing the auditory, visual, motor, oromotor/verbal, communication, and arousal processes. The lowest item on each subscale represents reflexive activity, whereas the highest item represents cognitively mediated behaviors.

### 2.4. EEG Data Recordings and Analysis

EEGs were recorded from 19 scalp positions using Ag/AgCl electrodes with a BrainAmp (Brain Products, Gilching, Germany) amplifier at a 500 Hz sampling rate with a notch filter at 50 Hz. The impedance of all the electrodes was kept below 10 k*Ω*. Scalp electrodes were positioned according to the international 10–20 system. All electrode sites were referenced online to FCz. A vertical electrooculogram (EOG) was recorded supraorbitally at the left eye. A horizontal EOG was recorded from the right orbital rim.

The EEG data were analyzed offline and processed using an average reference. Raw EEG data were digitally filtered between 0.5 and 40 Hz. A baseline correction was also applied to all channels. EEG epochs with ocular, muscular, and other artifacts were visually identified and manually rejected. Three conditions were selected for the analysis: EEGs at resting state acquired at T0, T1, and T2. A frequency spectrum was generated using the Welch's method (one of the Fourier transforms). The integral of different frequency bands on the frequency spectrum was used to obtain the absolute power, and the different frequency bands on the frequency spectrum were divided by 0.5–40 Hz to obtain the relative powers. Density power spectra were estimated for all frequencies between 0 and 512 Hz, and the relative power (%) was estimated for delta (1–4 Hz), theta (4–8 Hz), alpha (8–12 Hz), and beta (12–30 Hz) frequencies.

### 2.5. Statistical Analysis

First, to exclude the carryover effect (i.e., effect of the first phase influencing the second treatment period), differences in the CRS-R total score at the baseline of the two periods were compared using paired *t*-tests.

Second, to exclude the treatment order effect (i.e., a difference between the real-sham rTMS group and the sham-real rTMS group), the CRS-R scores variation at T1 and T2 were compared between the real-sham rTMS group and the sham-real rTMS group using Welch's unequal variances *t*-test.

If no carryover or order effects were shown, repeated measures analysis of variance (ANOVA) was applied to data to test the effect of treatment (real versus sham rTMS) at each time point on the CRS-R total score and the 6 CRS-R subscales scores with treatment and time as within-subject factors. When statistically significant differences (*α* = 0.05) were found in the main effects of time, post hoc Bonferroni correction for multiple comparisons were conducted; while the interaction of time and stimulation was significant, simple effects tests were followed.

The sphericity assumption was assessed using Mauchly's test before conducting repeated measures ANOVA. When the assumption was rejected, the Greenhouse-Geisser correction was used to adjust the degrees of freedom.

Finally, EEG data, both in real and sham rTMS stimulation conditions, were also compared with repeated measures analysis of variance (ANOVA) with Bonferroni corrections.

The statistical analysis was performed by SAS version 9.4 (SAS Institute Inc., Cary, NC). Statistical significance was set at *P* < 0.05.

## 3. Results

### 3.1. Clinical Effect: CRS-R

No significant differences were observed between the first and second stage of treatment indicated by the CRS-R total score at T0 (*P* = 0.363); therefore, the carryover effects of different periods were excluded. Additionally, we observed no order effects in the CRS-R score (T1: *P* = 0.423; T2: *P* = 0.374). The longitudinal change of CRS-R scores shows a slight improvement in response to both real and sham sessions; however, these changes were not significantly different on repeated measures ANOVA analysis (*P* = 0.376). Furthermore, no significant effect of rTMS was demonstrated on any of the six CRS-R subscales (*P* > 0.05).

At the individual level, one patient (number 4) showed a good clinical response to the real rTMS treatment at T1. The CRS-R total score changed from 6 to 8 and the CRS-R motor scoring changed from 1 to 3 (1 point: slow, stereotyped flexion or extension of the upper and/or lower extremities occurs immediately after the stimulus is applied; 3 points: the nonstimulated limb must locate and make contact with the stimulated body part at the point of stimulation), which denoted a MCS condition. And this effect lasted for one week after the stimulation (T2). Furthermore, another MCS patient (number 5) emerged from MCS at T2 during a sham rTMS session. None of the other patients showed any clinically remarkable response.

### 3.2. Neurophysiological Effects: EEG Reactivity

In these conditions, the carryover effects and order effects were excluded first (*P* > 0.05).

**Figure 2:**
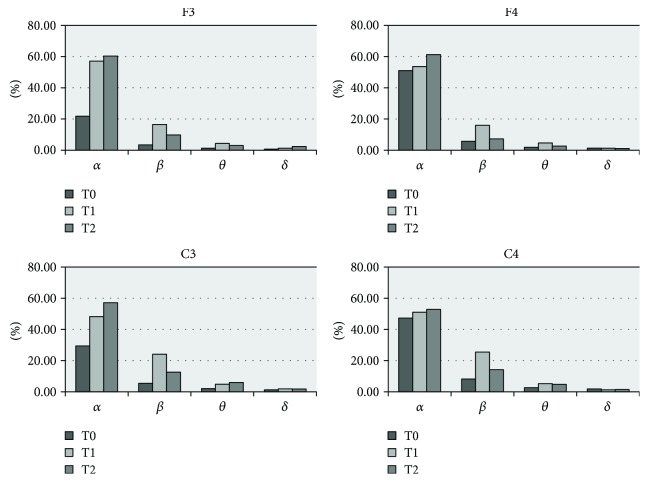
Delta, theta, alpha, and beta relative powers (%) prereal and postreal rTMS at T0 (beginning of the exam), T1 (after rTMS), and T2 (1 week after rTMS) in patient number 4. Almost all powers at F3, F4, C3, and C4 electrode sites increased after real rTMS treatment, especially at F3 and C3.

**Figure 3:**
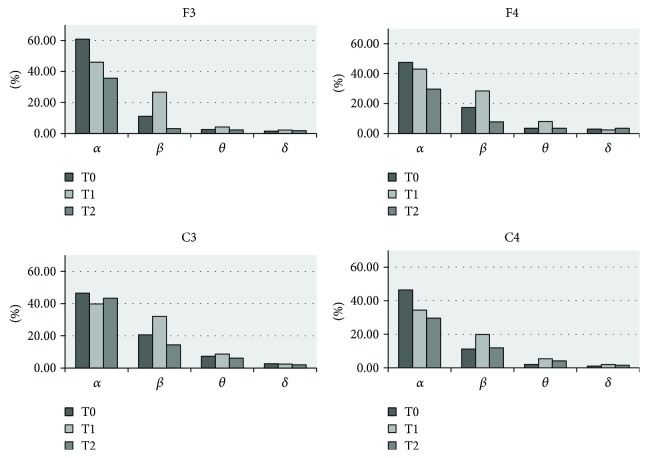
Delta, theta, alpha, and beta relative powers (%) presham and postsham rTMS at T0, T1, and T2 in patient number 5. The beta power at four electrode sites increased after sham rTMS treatment (T1) and decreased within one week (T2).

The clinical improvements of patient number 4 were accompanied by significant changes in the EEG power spectra. All four bands showed good EEG reactivity in response to real rTMS (except for C4 where the *δ* power decreased at both T1 and T2), especially at the F3 and C3 electrodes, and the power of *α* and *β* increased more significantly than *θ* and *δ*. Furthermore, the persistent improvement lasted for one week (Figure 2). However, the EEG power in patient number 5 was not significantly increased, only a transient improvement in *β* and *θ* range was observed. Notably, compared to the others, the EEG power at baseline (T0) was higher, particularly in the *α* band (Figure 3).

**Figure 4:**
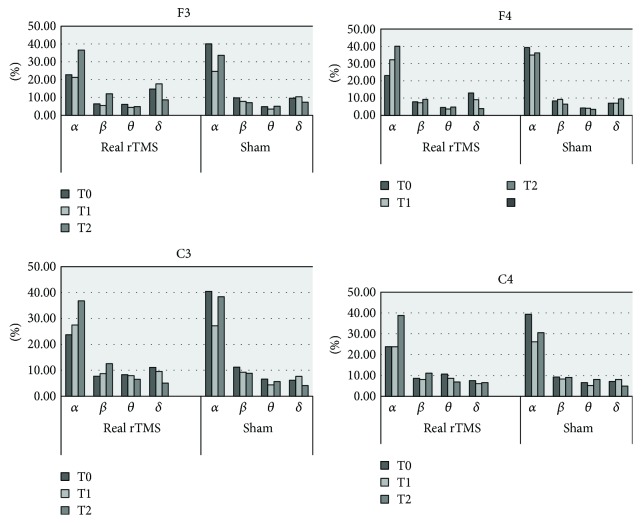
Grand average (5 patients, patients' numbers 1, 2, 3, 5, and 6) of delta, theta, alpha, and beta relative powers (%) prereal and postreal and presham and postsham rTMS at T0, T1, and T2.

Except for patient number 4, no other patient presented with any reliable, marked changes in EEG activity after transcranial stimulation at T1 or T2 (*P* < 0.05), although brain reactivity was incidentally found at several electrodes (*α* band at F4 and C3, *δ* band at F3, and *β* band at C3). One week following rTMS (T2), the power of *α* and *β* showed a trend towards an increase, although this did not reach significance (*P* > 0.05). As for sham rTMS, the EEG power tended to decrease after brain stimulation; however, this change was not statistically significant between T0 and T1 or T0 and T2 (*P* > 0.05, Figure 4).

## 4. Discussion

In this randomized, sham-controlled, rTMS clinical study in patients with DOC, high-frequency real or sham rTMS was administered to the left M1 of patients for 5 consecutive days. The results show that behavioral changes and EEG modifications were only observed in one VS patient. Another patient emerged from MCS but without EEG improvement. The remaining subjects did not show significant behavioral changes nor overall detectable EEG modifications.

At present, there have been few studies investigating the effect of rTMS on DOC, especially randomized, sham-controlled clinical studies. Our study showed a positive effect of rTMS in one VS patient. Conversely, the research of Cincotta et al. did not provide evidence for the efficacy of rTMS over M1 in VS treatment. Two hypotheses can be put forward to explain the difference observed between these two studies. First, EEG data were previously evaluated with Synek classification in five different grades. In our research, the EEG data were analyzed quantitatively using the power value, which is more sensitive to the detection of any subtle cortical activity. Second, the majority of VS patients in the previous study were anoxic-ischemic encephalopathy survivors with widespread brain injury with impaired functional connectivity among different brain areas. Furthermore, the CRS-R scores were 2 to 8 and the course of disease was more than 9 months. Based on the previous reviews and meta-analysis, a lower admission CRS-R score, longer duration, and anoxic encephalopathy predicted unfavorable functional outcome [22]. Recovery of function from a persistent VS, which is judged to be permanent 12 months after a traumatic injury and 3 months after a nontraumatic injury, is rare [23, 24]. The comparative analysis of these two studies suggests that the selection of eligible patients for rTMS would be conducive to the better allocation of medical resources.

In patient number 4, the observed clinical improvement was associated with EEG changes, particularly at sites F3 and C3. This matches previous reports [25]. When consciousness is reserved, the thalamocortical system should respond to TMS with a complex pattern of activation, involving various cortical areas; on the contrary, after loss of consciousness, TMS pulses only produce a simple activation that remains localized to the site of stimulation, indicating a breakdown of effective interactions among the thalamocortical modules [25]. Moreover, a PET study also indicated that VS patients show a cerebral response that is restricted to specific cortices in response to sensory or auditory stimulus [26].

Previous investigations have applied rTMS to the motor cortex and observed a transient increase of neuronal oscillations in the *α* and *β* frequency EEG band, whereas reactivity in the lowest frequency bands, *θ* and *δ*, is more prominent after magnetic stimulation of the dorsal premotor cortex than stimulation of the M1 [7, 27, 28]. This may explain why the *α* and *β* power increased significantly in the current study. Conversely, the proximity and connection between the M1 and the dorsal prefrontal cortex can increase the *θ* and *δ* bands. EEG activity is a symbol of the activation level of the brain cortex. The *α* range may be related to the cortical–thalamic interaction [28], whereas the oscillations are sensitive to the levels of GABA in the brain [29, 30]. Meanwhile, with 20 Hz rTMS stimulation, the increased release of dopamine may modulate the *δ* and *θ* activity [31], representing a phenomenon of rTMS-induced excitatory neuromodulation.

In this trial, it was notable that the resting EEG spectral power potentiation outlasted the one-week stimulation period. In unawakened patients, the effects of TMS were delayed with more obvious improvement recorded one week after stimulation compared with the improvement recorded immediately after a session of stimulation. Repetitive TMS, compared to single TMS, was more likely to produce long-lasting effects. We speculate that this may be related to brain plasticity. Numerous studies have unambiguously demonstrated that TMS signals stimulate and induce gene expression and enhance the production of a number of enzymes [32, 33]. These effects likely contribute to the long-lasting duration of the therapeutic effects of TMS with some changes only observable following rTMS. Additionally, a previous animal study indicated that 5 days of rTMS enhances BDNF binding affinity for TrkB, BDNF-TrkB signaling, and NMDA receptor-TrkB interaction in the rat prefrontal cortex [34]. The BDNF-TrkB system is an important upstream regulator of synaptic plasticity.

Comparing subjects number 4 and number 5, the latter showed improved clinical behavior but only a transient EEG activation at *β* and *θ* frequency. This result was in agreement with Johnson's research, in which rTMS influenced performance by biasing the endogenous task-related oscillatory dynamics, rather than creating “virtual lesion” with noise [35]. In our research, both real and sham rTMS produce noise, the real magnetic stimulation can not only produce a virtual lesion by injecting noise into the stimulated brain areas, but also can activate the deep brain structures, appearing as an EEG power potentiation.

When considering restorative mechanisms in DOC, thalamocortical pathways are implicated in rehabilitation [36]. The mesocircuit model hypothesizes that the highly dynamic and integrated thalamocortical network is driven by complex and synchronized neuronal firing patterns that are associated with the depolarization of cortical, thalamic, and striatal membrane potentials [37]. EEG recordings serve as a direct measure of neuronal activity. Such recordings have facilitated the integration of structural and functional cerebral changes, enabling a greater understanding of the variation between different states. Reviewing the EEG of subject number 5, the cerebral behavior was active at the baseline state. Thus, we speculate that it is on the way to modifications of the thalamocortical pathways and the CRS-R increased subsequently. As a result, the EEG response, more sensitive than CRS-R, is proposed as an early indicator of consciousness recovery.

However, there are some limitations in our research. The relatively small sample size limits the reliability of the therapeutic effects of rTMS. In the context of this enormous disability, controlled clinical trials (which may involve use of a placebo) are challenging to perform, because of the ethical issues linked to the severe nature of their clinical conditions and to the inability of the pessimistic subjects' legal guardians to provide informed consent. However, this study has provided evidence for the importance of appropriate patient selection. Nonanoxic individuals whose disease process is within 3 months are more likely to benefit from rTMS treatment. Another limitation of the current study is that the follow-up assessment and wash-out period were limited to one week. Although the carryover effect was excluded because it was not deemed statistically significant, it is possible that the prolonged effects may last more than one week following treatment; thus, it is useful to conduct follow-up testing at longer intervals. The third limitation of the current study is that the operator who delivered the stimulation was not blind to the type (real or sham) of rTMS application, so the performance bias could not be excluded. Nevertheless, the experimenter performing the clinical evaluations and analyzing the EEG data was blind to the study design. Considering the results from previous studies in conjunction with our current research, it cannot easily be confirmed that disease recovery can be attributed to the intervention. It is possible that the self-healing process may confuse the results. In our study, we abandoned potential “awakening” drugs (GABAergic and monoaminergic drugs) [38], and two clinicians performed a daily assessment for 15 days, at different times, before the study enrollment in order to assess a truly and stable condition. Nonetheless, in view of the strong subjectivity and the weakness in detecting subtle behavioral improvement with CRS-R, the sensitive and scientific EEG method is also recommended for use in disease evaluation in future.

## Figures and Tables

**Table 1:** Clinical data of patients with DOC.

Patient	Age	Gender	Clinical diagnosis	Etiology	MRI findings	Month since injury
P1	49	F	VS	Traumatic	DAI and SAH	4
P2	14	M	MCS	Hypoxic-ischemic	Diffuse demyelination	4
P3	45	F	VS	Traumatic	Bilateral temporal and occipital lobe lesions	28
P4	58	M	VS	Hemorrhagic	Right basal ganglion, thalamus, and corpus callosum lesions	2
P5	42	M	MCS	Traumatic	Right frontal, temporal, and parietal lobe and bilateral cerebellum and corpus callosum lesions	1
P6	29	M	EMCS	Traumatic	Bilateral frontal, temporal lobe and brain stem lesions	10

**Table 2:** DOC subjects' data at the CRS-R score.

	Real rTMS			Sham		
	T0	T1	T2	T0	T1	T2
P1	7 (1, 1, 2, 1, 0, 2)	7 (1, 1, 2, 1, 0, 2)	7 (1, 1, 2, 1, 0, 2)	7 (1, 1, 2, 1, 0, 2)	7 (1, 1, 2, 1, 0, 2)	7 (1, 1, 2, 1, 0, 2)
P2	16 (4, 5, 2, 1, 1, 3)	16 (4, 5, 2, 1, 1, 3)	15 (4, 5, 2, 1, 1, 2)	16 (4, 5, 2, 1, 1, 3)	16 (4, 5, 2, 1, 1, 3)	16 (4, 5, 2, 1, 1, 3)
P3	7 (1, 1, 2, 1, 0, 2)	7 (1, 1, 2, 1, 0, 2)	7 (1, 1, 2, 1, 0, 2)	7 (1, 1, 2, 1, 0, 2)	7 (1, 1, 2, 1, 0, 2)	7 (1, 1, 2, 1, 0, 2)
P4	6 (1, 1, 1, 1, 0, 2)	8 (1, 1, 3, 1, 0, 2)	8 (1, 1, 3, 1, 0, 2)	6 (1, 1, 1, 1, 0, 2)	6 (1, 1, 1, 1, 0, 2)	6 (1, 1, 1, 1, 0, 2)
P5	23 (4, 5, 6, 3, 2, 3)	23 (4, 5, 6, 3, 2, 3)	23 (4, 5, 6, 3, 2, 3)	15 (2, 4, 4, 2, 1, 2)	15 (2, 4, 4, 2, 1, 2)	23 (4, 5, 6, 3, 2, 3)
P6	23 (4, 5, 6, 3, 2, 3)	23 (4, 5, 6, 3, 2, 3)	23 (4, 5, 6, 3, 2, 3)	23 (4, 5, 6, 3, 2, 3)	23 (4, 5, 6, 3, 2, 3)	23 (4, 5, 6, 3, 2, 3)

CRS-R = coma recovery scale-revised; six subscales score of CRS-R indicating the assessment of auditory, visual, motor, verbal, communication functions, and arousal.
